# Supplementary material for: Pathfinder: open source software for analyzing spatial navigation search strategies
Source: F1000Res. 2020 Jun 15;8:1521. Originally published 2019 Aug 28. [Version 2] doi: 10.12688/f1000research.20352.2 (PMC6974928; doi:10.12688/f1000research.20352.2)
Supplement: Peer review history

# Peer review: Reviewer response for version 2

**DOI:** 10.5256/f1000research.27222.r64767

**Published:** 2020-07-10

**Authors:** Matthew Holahan

**Affiliations:** 1 Department of Neuroscience, Carleton University, Ottawa, ON, Canada

I have no concern with Version 2.

Are the conclusions about the tool and its performance adequately supported by the findings presented in the article?

Yes

Is the rationale for developing the new software tool clearly explained?

Yes

Is the description of the software tool technically sound?

Yes

Are sufficient details of the code, methods and analysis (if applicable) provided to allow replication of the software development and its use by others?

Yes

Is sufficient information provided to allow interpretation of the expected output datasets and any results generated using the tool?

Yes

Reviewer Expertise:

Spatial learning and memory, Parkinson's disease, concussions, development, hippocampus, addiction

I confirm that I have read this submission and believe that I have an appropriate level of expertise to confirm that it is of an acceptable scientific standard.

# Peer review: Reviewer response for version 1

**DOI:** 10.5256/f1000research.22365.r56564

**Published:** 2020-01-21

**Authors:** Robert McDonald, Nancy S. Hong

**Affiliations:** 1 Department of Neuroscience, Canadian Center for Behavioural Neuroscience, Lethbridge University, Lethbridge, AB, Canada; 2 Department of Neuroscience, University of Lethbridge, Lethbridge, Canada

**Summary of the contribution:**

 A recent paper submitted by Snyder and colleagues to F1000Research concerns the use of the Morris water task as an assay of learning and memory functions in the mammal. As noted 10 years ago, this is probably the most widely used assay of both spatial navigational processes and cognitive functions, in the rodent, in the history of psychology and behavioural neuroscience ^1^. 

 This paper introduces an open source analysis program, “Pathfinder”, for assessing the traditional measures of spatial learning and memory, and also an evaluation of swim patterns/strategies that change in a systematic way during different stages of learning. The paper provides a brief historical account of the discovery and use of the Morris water task (MWT) suggesting that the earlier work lacked sophistication in their analysis of this complex cognitive/navigational behaviour, relying mostly on latency and path length. This is followed by a description of the new software including that it easily interfaces with commonly used tracking and behavioural analysis systems, and that it will be available for free. Both of these features will ensure that it can be used widely by scientists with varying ideas and views. Importantly, the authors provide “proof of principle” experiments highlighting its strengths at assessing the classic measures of place learning and memory, as well as the newer assessment of swim patterns during the different stages of acquisition and retention. The graphical user interface is excellent and allows the experimenter to determine the particular parameters of interest. A nice addition to this part of the paper was the verification of the data analysis completed by the new system as compared to several human observers, showing a high level of agreement. We also believe that having this software available to anyone interested in learning and memory processes in a variety of less constrained open-field situations will be very beneficial. 

 We would like to raise some issues for the readers and authors to think about relating to historical context, including early uses of swim pattern analysis, potential benefits and pitfalls of the swim pattern analysis, the nature of the representation acquired during task acquisition, and the possibility of future advances that might prove impactful. 

**Historical context:**

 It is important for the reader interested in using the MWT as a measure of learning and memory processes to get a better view of the history and development of the task which will place the new “Pathfinder” system in historical context. This exposition points to various empirical issues and theoretical questions that could be solved with future developments of the program. For example, the contributions of the “Lethbridge group” should be noted. In particular, Sutherland along with Whishaw and Kolb published some of the earliest lesion work using this task ^2^^,^^3^. During this time, there was probably a significant amount of communication (see earliest papers and conference abstracts) between Morris and the Lethbridge group concerning the effects of fornix lesions, hippocampal ablations, and ultimately neurotoxins on the place learning version of this task. Sutherland went on to do important work using this paradigm investigating the subcortical and neocortical circuits implicated in this complex form of spatial navigation ^4^^,^^5^. The Lethbridge group also contributed significantly to our understanding of the contributions of different neurotransmitters ^6^, the important distinction between motor versus cognitive impairments ^7^, the nature of the representation acquired during training ^8^^,^^9^, etc. 

 The role of the HVS tracking system in making the MWT such an important tool for behavioural neuroscience and biomedical research should also not be forgotten. The two key labs (Morris and Sutherland) that contributed so much to the development of this task and understanding the brain systems and mechanisms involved used the HVS tracking system and then wrote their own data collection and analysis programs for use in their respective labs. 

 We also found in the paper, the general tendency to suggest that the early work was quite primitive and focused on latency and path length. I do not think this is accurate. If one looks at the early work by both groups, there was clear use of multiple parameters (latency, path length, heading angle, quadrant preference, thigmotaxia, rearing on the platform, wall and start-point revisits, and annulus crossings). This work was followed by assessment of different strategies used by rats following brain manipulations while acquiring the MWT. For example, rats with HPC lesions can show one successful strategy in which they learn to circumnavigate a certain distance away from the pool wall and run into the escape platform ^10^. As well, other strategy changes have also been described following specific neuropharmacological manipulations ^11^. The annulus crossings measure is interesting because it shows an early interest in spatial specificity of the place responses acquired following training on the MWT. The importance of this measure for understanding the neural basis of learning on this task was not clear at the time, but became evident when we were involved in addressing controversies over whether the HPC was important for learning this task or not ^12^^,^^13^. The spatial specificity measure was an important part of our rebuttals to these claims ^14^. Accordingly, we take significant credit for the idea that rats with HPC dysfunction can show a “place response” under some training conditions, but they do not show spatial specificity (but see Kolarik *et al*., 2018 ^15^). 

 Devan and colleagues ^16^^,^^17^ also used a swim path analysis to counter claims ^18^ that the cause of impairments on the standard, spatial version of the water task, induced by brain manipulations (lesions, pharmacology, etc) were due to partial reinforcement effects and not because the subjects were unable to learn and remember the location of the escape platform. According to Gonzalez and colleagues ^18^, the deficits in these experimental subjects were caused by the brain manipulations disrupting the supports of learning and memory (sensory, motor, and motivation) that resulted in the subjects finding the platform fewer times during training than a normal subject. We assessed these serious claims and showed that normal rats that received partial reinforcement on the spatial version of the water task showed impaired escape latencies. However, using a combination of time bin and zone analysis, we showed that the partially reinforced group went directly to the correct spatial location early in the trial and when they did not find it, swam to the pool wall. Note that the pool wall is also a source of reinforcement because the subject is removed by the experimenter at this barrier when a trial ends. Based on our analysis we concluded that, even under partial reinforcement training conditions, control rats learn the precise location of the escape platform, but other reinforcement contingencies also have an influence on their subsequent behaviour as the experimenter is also associated with escape from the pool.

 We have also shown that rats can sequentially learn two different escape locations and during a probe test visit the most recent location first and then the old location ^19^. It would be interesting if the authors discussed how the “Pathfinder” system could be used to capture these complex sets of swim strategies, in the different experiments described, and how bin analyses could be incorporated. 

 The authors of the current paper also describe vicarious trial and error (VTE) like behaviours exhibited when multiple locations are in play at the same time. In our experience, the most compelling demonstration of these kinds of competition-like behaviours is during the final test day following 12 days of cue/place learning ^20^. When the cued platform was moved to a new spatial location and the subjects were started from an equidistant point between the old spatial location and the new cue location, some of the rats went to the new cue position and the others went to the old spatial location first. Interestingly, watching videos of the subjects during this competition test revealed some interesting patterns of behaviour. Some went directly to the cue, some went directly to the place, others started heading to one platform position and then switched direction, and some would change course several times of move their head back and forth to go back and forth before making a decision. We imagine this emerging when representations acquired by two parallel learning and memory networks are “flickering” back and forth in controlling the subject’s behaviour ^21^. Again, it is not clear from the manuscript how “Pathfinder” would pick up these kinds of micro-behaviours or the nature of further advancements in the program that might accommodate these more complex learning situations. Maybe a further expansion of this would be helpful in the manuscript.

**Potential benefits and pitfalls of the automated swim path analysis approach:**

 As noted by the authors, it is not clear what the potential benefits of the swim path analysis produced by the “Pathfinder” system might be, and they provide some possible lines of research that might be fruitful. In our view, one area of enquiry that might be particularly fruitful is a reassessment of the learning and memory network centered on the HPC and the role of each of these areas (HPC, fornix, subiculum, thalamus, nucleus accumbens, mammillary bodies, prefrontal cortex, cingulate cortex, etc) to the different components of place navigation (strategies and strategy transitions). Another potentially interesting area of enquiry would be to revisit the contributions of various neurotransmitters including the ascending modulator systems as well as neurotransmitter systems localized in each sub-region. These lines of enquiry could be developed using both traditional as well as “state of the art” techniques including neurotoxic lesions, inactivation techniques, behavioural pharmacology, optogenetics, imaging, etc.

 Our experience with swim path analysis by human observers and comparing it to those generated by automated versions have been interesting. In a recent paper ^22^, we assessed swim path strategies and transitions during the course of training on the spatial version of the water task based on early classic water task experiments and the approach taken by Ruediger and colleagues ^23^. We assessed the navigation response strategy used by rats during all training trials by viewing each swim and categorizing the behaviour. We replicated their findings that mice with ventral HPC lesions are impaired early in training and not later in training. Importantly, we also discovered some clear differences that are relevant to the “Pathfinder” program. In our experiment, the VHPC lesioned rats used general random search strategies in the early and middle stages of training, while the sham rats switched to direct search strategies towards the escape platform earlier. This switch to direct search strategies by control rats was earlier than control mice in the Ruediger experiments. As we have argued before, these kinds of species and potentially strain differences are important to be aware of when using these kinds of automated analysis systems ^22^.

 We found other potential caveats associated with these automated approaches. For example, for some individual swims, the criterion for a specific strategy either: did not categorize the subject’s behaviour accurately; some swims could be categorized incorrectly; or no categorization fit the swim pattern exhibited. One of the most interesting examples we came across was late in training in which the swim strategies would be described as hybrid strategies. These include: thigmotaxia early in the swim and then a quick turn to the platform when the subject was close to the correct area of the escape location; circumnavigation but in zones A and B (HVS image); looping but with most time in zone A; single short looping behaviours to escape platform. The point of this simple analysis is that we understand the advantages of using automated algorithms for categorizing swim navigational strategies, but they have some disadvantages including missed strategies or swims categorized incorrectly. We strongly advocate that anytime a lab employs a new experimental manipulation (brain perturbation, strain, sex, age, etc) that swim navigational strategies are assessed with both automated and human observers. 

**Nature of the representations used to navigate in the Morris water task:**

 One final issue that is relevant to the “Pathfinder” analysis program is that it remains a bit of a mystery as to what the actual representation(s) and associated networks that guide behaviour during acquisition and retention of the spatial version of the water task. 

 Based on some experiments using the MWT ^9^, it appears, somewhat counterintuitively that rats use the shape of the testing apparatus and directional information to locate the escape location. For these experiments, rats were trained on the standard, spatial version of the water task and then a probe trial was completed in which the maze was moved in the training context in a way that allowed the rats to either try to locate the escape platform via directional information or absolute spatial location information. The results clearly showed that a majority of the rats used a directional representation and not a representation of the relationships among the cues in the testing room. Our follow-up experiments assessing the implications of this work extended these results and showed that this directional representation seems to be based on pool shape information combined with head direction information encoded by the post-subiculum and related head direction network ^23^. Even more striking, this directional representation transfers to new contexts, but the behaviours supported by this representation would be deleterious to performance unless the experimenter places the platform in the location based on directional information regardless of which context they are navigating. This is important to be aware of for various reasons, but particularly relevant in the current review because transferring to a new room will not result in random searches of the pool, as generally assumed. Rather, early on in training they will be swimming to a location that is now probably incorrect.

 Finally, we implore researchers to ensure that during crucial probe (transfer) tests that the experimenter stir the pool water after each swim to ensure that olfactory cues are not supporting the place response ^13^.

**Conclusions**

 The Pathfinder program should prove to be a welcome addition to the toolbox available to researchers interested in the learning and memory processes involved in place navigation and the neural networks and mechanisms supporting these complex functions. Making it more accessible for scientists to look at various swim patterns during different stages of training will inevitably make it more mainstream to do so. In our view, further advancements to this new approach will increase its impact substantially.

Are the conclusions about the tool and its performance adequately supported by the findings presented in the article?

Yes

Is the rationale for developing the new software tool clearly explained?

Yes

Is the description of the software tool technically sound?

Yes

Are sufficient details of the code, methods and analysis (if applicable) provided to allow replication of the software development and its use by others?

Yes

Is sufficient information provided to allow interpretation of the expected output datasets and any results generated using the tool?

Yes

Reviewer Expertise:

Neural basis of learning and memory.

We confirm that we have read this submission and believe that we have an appropriate level of expertise to confirm that it is of an acceptable scientific standard.

## References

[1] : The challenges of understanding mammalian cognition and memory-based behaviours: an interactive learning and memory systems approach. *Neurosci Biobehav Rev*.2004;28(7) : 10.1016/j.neubiorev.2004.09.014 719-45 10.1016/j.neubiorev.2004.09.014 15555681

[2] : Spatial mapping: definitive disruption by hippocampal or medial frontal cortical damage in the rat. *Neurosci Lett*.1982;31(3) : 10.1016/0304-3940(82)90032-5 271-6 10.1016/0304-3940(82)90032-5 7133562

[3] : A behavioural analysis of spatial localization following electrolytic, kainate- or colchicine-induced damage to the hippocampal formation in the rat. *Behav Brain Res*.1983;7(2) : 10.1016/0166-4328(83)90188-2 133-53 10.1016/0166-4328(83)90188-2 6830648

[4] : The role of the fornix/fimbria and some related subcortical structures in place learning and memory. *Behav Brain Res*.1989;32(3) : 10.1016/s0166-4328(89)80059-2 265-77 10.1016/s0166-4328(89)80059-2 2496702

[5] : Contributions of cingulate cortex to two forms of spatial learning and memory. *J Neurosci*.1988;8(6) :1863-72 338547810.1523/JNEUROSCI.08-06-01863.1988PMC6569344

[6] : Cholinergic receptor blockade impairs spatial localization by use of distal cues in the rat. *J Comp Physiol Psychol*.1982;96(4) : 10.1037/h0077914 563-73 10.1037/h0077914 7119176

[7] : Cholinergic receptor blockade produces impairments in a sensorimotor subsystem for place navigation in the rat: evidence from sensory, motor, and acquisition tests in a swimming pool. *Behav Neurosci*.1987;101(5) : 10.1037//0735-7044.101.5.603 603-16 10.1037//0735-7044.101.5.603 3675838

[8] : Hippocampal lesions and path integration. *Curr Opin Neurobiol*.1997;7(2) : 10.1016/s0959-4388(97)80011-6 228-34 10.1016/s0959-4388(97)80011-6 9142750

[9] : How do room and apparatus cues control navigation in the Morris water task? Evidence for distinct contributions to a movement vector. *J Exp Psychol Anim Behav Process*.2007;33(2) : 10.1037/0097-7403.33.2.100 100-14 10.1037/0097-7403.33.2.100 17469959

[10] : Place navigation by rats in a swimming pool. *Canadian Journal of Psychology/Revue canadienne de psychologie*.1984;38(2) : 10.1037/h0080832 322-347 10.1037/h0080832

[11] : Disruption of central cholinergic systems in the rat by basal forebrain lesions or atropine: effects on feeding, sensorimotor behaviour, locomotor activity and spatial navigation. *Behav Brain Res*.17(2) : 10.1016/0166-4328(85)90023-3 103-15 10.1016/0166-4328(85)90023-3 4074488

[12] : Rats with fimbria-fornix lesions display a place response in a swimming pool: a dissociation between getting there and knowing where. *J Neurosci*.1995;15(8) :5779-88 764321810.1523/JNEUROSCI.15-08-05779.1995PMC6577651

[13] : Evidence for extrahippocampal involvement in place learning and hippocampal involvement in path integration. *Hippocampus*.1996;6(5) : 10.1002/(SICI)1098-1063(1996)6:5<513::AID-HIPO4>3.0.CO;2-J 513-24 10.1002/(SICI)1098-1063(1996)6:5<513::AID-HIPO4>3.0.CO;2-J 8953304

[14] : Rats with hippocampal damage are impaired on place learning in the water task when overtrained under constrained conditions. *Hippocampus*.2000;10(2) : 10.1002/(SICI)1098-1063(2000)10:2<153::AID-HIPO4>3.0.CO;2-A 153-61 10.1002/(SICI)1098-1063(2000)10:2<153::AID-HIPO4>3.0.CO;2-A 10791837

[15] : Close but no cigar: Spatial precision deficits following medial temporal lobe lesions provide novel insight into theoretical models of navigation and memory. *Hippocampus*.28(1) : 10.1002/hipo.22801 31-41 10.1002/hipo.22801 28888032PMC5747326

[16] : Partial reinforcement across trials impairs escape performance but spares place learning in the water maze. *Behav Brain Res*.2003;141(2) : 10.1016/s0166-4328(02)00294-2 91-104 10.1016/s0166-4328(02)00294-2 12742245

[17] : A cautionary note on interpreting the effects of partial reinforcement on place learning performance in the water maze. *Behav Brain Res*.2001;119(2) : 10.1016/s0166-4328(00)00342-9 213-6 10.1016/s0166-4328(00)00342-9 11165337

[18] : A cautionary note regarding drug and brain lesion studies that use swimming pool tasks: partial reinforcement impairs acquisition of place learning in a swimming pool but not on dry land. *Behav Brain Res*.2000;112(1-2) : 10.1016/s0166-4328(00)00162-5 43-52 10.1016/s0166-4328(00)00162-5 10862934

[19] : NMDA-receptor blockade by CPP impairs post-training consolidation of a rapidly acquired spatial representation in rat hippocampus. *Eur J Neurosci*.2005;22(5) : 10.1111/j.1460-9568.2005.04272.x 1201-13 10.1111/j.1460-9568.2005.04272.x 16176363

[20] : Parallel information processing in the water maze: evidence for independent memory systems involving dorsal striatum and hippocampus. *Behav Neural Biol*.1994;61(3) : 10.1016/s0163-1047(05)80009-3 260-70 10.1016/s0163-1047(05)80009-3 8067981

[21] : Context, emotion, and the strategic pursuit of goals: interactions among multiple brain systems controlling motivated behavior. *Front Behav Neurosci*.2012;6: 10.3389/fnbeh.2012.00050 50 10.3389/fnbeh.2012.00050 22876225PMC3411069

[22] : Rats with ventral hippocampal damage are impaired at various forms of learning including conditioned inhibition, spatial navigation, and discriminative fear conditioning to similar contexts. *Behavioural Brain Research*.2018;351: 10.1016/j.bbr.2018.06.003 138-151 10.1016/j.bbr.2018.06.003 29883593

[23] : Goal-oriented searching mediated by ventral hippocampus early in trial-and-error learning. *Nat Neurosci*.2012;15(11) : 10.1038/nn.3224 1563-71 10.1038/nn.3224 23001061

# Response

**Published:** 2020-06-06

**Authors:** Jason Snyder

**Affiliations:** Hospital for Sick Children, Canada

Robert and Nancy – thank you for your detailed and thoughtful review. The additional background information and interpretations will be useful for those wishing to use Pathfinder when designing and analyzing their water maze experiments. We generally agree with the points you raise and would like to comment on a few of them specifically.

We understand that it may have sounded like we imply that early water maze work was primitive. We would like to clarify that we in fact tried to emphasize the opposite! Our take is similar to yours, which is that the early work by Morris, Sutherland, Whishaw etc performed quite extensive analyses which were essential to validate the water maze. Ultimately, as behavioral testing has become much more common, and since latency and path length are decent measures of basic performance, they soon dominated and detailed analyses became less common. Of course, even the early work did not conduct strategy analyses that are comparable to those offered by Pathfinder, but certainly the quality of those original papers cannot be understated. We have made some minor adjustments to the text to clarify our view.

You also raise some questions about how Pathfinder could be employed to detect strategies related to competing goals. We attempted to describe a few situations where rodents are tasked with deciding between spatial vs cued goals and multiple possible spatial goals. We cite these papers for the readers’ reference and feel that these provide a good illustration of how the water maze can be used to study decision-making , conflict between multiple strategies/brain regions and the like. But the possibilities are endless and so ultimately it will depend on the creativity of the users.

We appreciate your comments about the importance of validating automated software with manual analyses. We agree that this is critical at the outset. Only when the user trusts their parameter settings should they proceed with the full analysis.

Finally, the issue of hybrid strategies is one that Pathfinder currently cannot address, unless one is willing to bin their trials prior to importing into Pathfinder. For a hybrid approach one may want to try the software described in Vourous, 2018, Scientific Reports since it can detect multiple strategies in a single trial.

# Peer review: Reviewer response for version 1

**DOI:** 10.5256/f1000research.22365.r56738

**Published:** 2019-12-02

**Authors:** Matthew Holahan

**Affiliations:** 1 Department of Neuroscience, Carleton University, Ottawa, ON, Canada

I agree with the authors that the assessment of spatial information processing in rodents with automatized tracking systems aids in the reliability of findings across studies and enhances efficiency in which one can make conclusions about specific manipulations. The current work, which would have been nice to include, would be more complete with:

1. A direct comparison to commercially available software - e.g., HVS Image provides similar tracking information so in this case, how do these two tracking systems fair head to head? 
2. A group of mice with hippocampal lesions. 

For point 2, does the tracking software pick out well-established deficits in spatial behavior following hippocampal lesions and does the new software provide any additional information on the deficits in spatial navigation produced by hippocampal lesions. I wonder also if the additional measures provided by this software will give more insight into spatial learning deficits in models where deficits were not noted; that is to say, conventional tracking software or output measures might not revealed a deficit when a deficit was predicted. Would this new software provide more robust measures to indicate deficits? Further work is required to more fully test the software to demonstrate its reliability.

Are the conclusions about the tool and its performance adequately supported by the findings presented in the article?

Yes

Is the rationale for developing the new software tool clearly explained?

Yes

Is the description of the software tool technically sound?

Yes

Are sufficient details of the code, methods and analysis (if applicable) provided to allow replication of the software development and its use by others?

Yes

Is sufficient information provided to allow interpretation of the expected output datasets and any results generated using the tool?

Yes

Reviewer Expertise:

Spatial learning and memory, Parkinson's disease, concussions, development, hippocampus, addiction

I confirm that I have read this submission and believe that I have an appropriate level of expertise to confirm that it is of an acceptable scientific standard.

# Response

**Published:** 2020-06-06

**Authors:** Jason Snyder

**Affiliations:** Hospital for Sick Children, Canada

Matthew – thank you for the feedback. Our response is a little delayed, because we have timed it to coincide with a software/manuscript update, which took longer than anticipated.

With respect to your points, we were unaware that any commercial software packages offer strategy-based analyses. There are some other free programs created by researchers, and it would definitely be interesting to do a comparison. It certainly would have been nice to validate it using an established model of hippocampal deficits, like a lesion. I do think it would pick up deficits that have been described, since hippocampal-lesioned rodents are typically thigmotactic and less spatially accurate, which Pathfinder clearly captures in the early stages of training in our experiment. We have validated it with neurogenesis-deficient rats (Yu, Behav Brain Res, 2019), where these rats displayed a strategy phenotype that was probably relatively subtle compared to hippocampal-lesioned animals. Finally, I do think it could be used to detect behavioral changes that other programs cannot. For this to work, it will be important for the user to understand their subject’s behavior and adjust the parameters accordingly such that the strategies are captured. This will increase strategy detection accuracy and sensitivity but users always need to be cautious when interpreting the output, and assess whether finely-tuned parameters are detecting meaningful differences or not. In any case, there should be lots of existing navigational datasets out there that could be run with Pathfinder to address these exact questions. We look forward to seeing what people find.
